# Efficacy of Peginterferon Alpha-2a vs. 2b for the Treatment of Chronic HCV: Interpretation of Results and Future Prospects

**Published:** 2010-09-01

**Authors:** Hossein Khedmat, Saeed Taheri

**Affiliations:** 1 Baqiyatallah Research Center for Gastroenterology and Liver Diseases, Baqiyatallah University of Medical Sciences, Tehran, Iran; 2 Dr.Taheri Medical Research Group, Tehran, Iran

Dear Editor,

In their meta-analysis of the efficacy of peginterferon alfa-2a (PEG-IFN-α2a) or alfa-2b (PEG-IFN-α2b) and ribavirin for the treatment of hepatitis C virus (HCV), Alavian et al. [1], analyzing pooled data from 7 studies, conclude that PEG-IFN-α2a (with similar safety) is more effective than PEG-IFN-α2b. While PEG-IFN alfa-2b with ribavirin is generally considered as the treatment of choice for chronic HCV infection [2], the findings of this study sound at first like good news, especially since PEG-IFN alfa-2a is more cost-effective than PEG-IFN alfa-2b [3]; but we suggest that the findings of this study should be open to a more conservative interpretation.Because of the nature of meta-analysis, we cannot expect in the current article to consider all the major factors that may interfere or otherwise affect the efficacy or safety of PEG-IFNs. In Alavian's study, it was well demonstrated that PEG-IFN alfa-2a provides a higher virologic response than that of alfa-2b. However, several factors that can interfere, such as insulin resistance, racial factors (Asian patients respond better to PEG-IFN, but there is a greater incidence of adverse events among them), gender effect (females respond better to therapy), in addition to some genetic factors, were not included in this analysis; moreover, the study would have been more informative if genotype 1 of HCV were analyzed separately, since this genotype represents the most resistant type of HCV infection.A higher prevalence of neutropenia among patients treating with PEG-IFN alfa-2a was another interesting finding of the previously mentioned meta-analysis. Here we raise a question about the logic behind the disparities found: Can these observations be explained by a dose-response effect? McHutchison et al. [4] in their study found that a higher dosage of PEG-IFN alfa-2b is associated with a higher percentage in sustained virologic response (SVR), while a lower dosage was associated with a substantial decrease in neutropenia and anemia. According to these findings, we might be able to simply conclude that administration of a higher dosage of PEG-IFN alfa-2b may result in a higher SVR rate as well as neutropenia, comparable to that of PEG-IFN alfa-2a.McHutchison et al. [4], whose data comprise over 40% of the whole population in this meta-analysis, found that the relapse rate was substantially lower for PEG-IFN alfa-2b than for alfa-2a; another interesting observation of their study was that a lower dosage of PEG-IFN alfa-2b was associated with a lower relapse rate. The relapse rate was an important issue that was inevitably not considered in this study and needs to be addressed in future studies.Virologic response, although an important marker of a disease's progression and even of histological condition, is not exactly what we are concerned about; long-term prognosis such as improvement in survival as well as a decrease in transplantation rate is what preoccupies us, and we need to confirm the efficacy of using both of these two agents in clinical practice. There is no evidence that virologic response would lead to a better outcome; on the other hand, unfortunately, there is a shortage of data on these hard end points in the literature. We therefore recommend that this issue be urgently addressed in future studies.
